# Differential expression of serum mir-363-3p in patients with polycystic ovary syndrome and its predictive value for their pregnancy

**DOI:** 10.1186/s12905-023-02337-9

**Published:** 2023-05-15

**Authors:** Min Sang, Ying Yu, Zhi Zhou, Yaqi Zhang, Haiping Chang

**Affiliations:** 1 Reproductive Medicine Center, Huanggang Central Hospital of Yangtze University (Dabie Mountain Regional Medical Center), Huanggang, 438000 Hubei P.R. China; 2 Scientific Research and Teaching Department, Huanggang Central Hospital of Yangtze University (Dabie Mountain Regional Medical Center), Huanggang, 438000 Hubei P.R. China; 3 Department of Gynecology, Huanggang Central Hospital of Yangtze University (Dabie Mountain Regional Medical Center), No.126 Qi’an Avenue, Huangzhou District, Huanggang, 438000 Hubei P.R. China

**Keywords:** Polycystic ovarian syndrome, Sex hormone, MiR-363-3p, Pregnancy outcome

## Abstract

**Background:**

This study aimed to investigate the expression of serum miR-363-3p in patients with polycystic ovary syndrome (PCOS) and its predictive value for pregnancy after ovulation induction therapy.

**Methods:**

The expression of serum miR-363-3p was detected by Reverse transcription quantitative polymerase chain reaction (RT-qPCR). PCOS patients were treated with ovulation induction therapy, and after the successful pregnancy was confirmed, they were followed up for 1 year in outpatient department to record the pregnancy outcomes of the patients. The Pearson correlation coefficient was used to evaluate the correlation between the expression level of miR-363-3p and biochemical indicators of PCOS patients. Logistic regression analysis was used to analyze the risk factors of pregnancy failure after ovulation induction therapy.

**Results:**

The serum level of miR-363-3p in PCOS group was significantly lower than that in control group. Compared with the control group, both pregnant and non-pregnant groups had lower miR-363-3p levels, while the non-pregnant group had a greater reduction in miR-363-3p levels than the pregnant group. Low levels of miR-363-3p showed high accuracy in distinguishing pregnant and non-pregnant patients. Logistic regression analysis showed that high levels of luteinizing hormone, testosterone (T), prolactin (PRL) and low level of miR-363-3p were independent risk factors for pregnancy failure after ovulation induction in PCOS patients. Additionally, compared with pregnancy outcomes of healthy women, the incidence of premature delivery, macrosomia, and gestational diabetes in PCOS patients increased.

**Conclusions:**

The expression of miR-363-3p in PCOS patients was reduced and correlated with abnormal hormone levels, suggesting that miR-363-3p may be involved in the occurrence and development of PCOS.

## Background

Polycystic ovary syndrome (PCOS) is a common endocrine system disease in women of childbearing age, and it is also a research hotspot and focal issue in the entire field of clinical reproductive medicine [1]. There are some individual differences in PCOS patients, but the clinical manifestations are mainly concentrated in three aspects of sparse ovulation, polycystic ovarian changes and hyperandrogenism [2]. According to research statistics, the incidence of PCOS in women of childbearing age is about 10%, and infertility caused by PCOS accounts for about 60% of all infertile patients [3]. With the in-depth study of PCOS, people have also discovered the long-term impact of PCOS on women. In fact, PCOS can not only affect the women’s reproductive function, but also lead to metabolic disorders, such as hyperinsulinemia and insulin resistance, thus increasing the risk of secondary diabetes, cardiovascular disease, endometrial cancer, which has a negative impact on women’s health [4, 5].

MicroRNAs are a kind of non-coding RNAs with a length of 18–24 nucleotides, which is widely distributed in eukaryotes and usually play a negative regulatory role in vivo [6]. In recent years, there are more and more studies on miRNA in PCOS, and the research samples include serum, follicular fluid, ovarian granulosa cells and PCOS animal models [7]. MiRNA family is large and numerous. At present, only part of miRNA has been explored in PCOS. After analyzing the data of granular cell microarray in PCOS patients, Wang et al. found that miR-486-5p was involved in regulating the growth and development of follicles [8]. Another study found that there were differentially expressed miRNAs in follicular fluid, such as miR-135 and miR-32, which are potential new biomarkers of PCOS [9]. MiR-363-3p, located on the human X chromosome, is one of the members of the miR-92a family. At present, the function of miR-363-3p mainly focused on tumor immunity [10]. Studies have shown that miR-363-3p can promote the occurrence of gastric cancer by down-regulating the expression of tumor suppressor gene MBP-1 [11]. In addition, the level of miR-363-3p in vivo is related to the invasion and metastasis of head and neck squamous cell carcinoma by acting on tumor invasion and metastasis-related proteins [12]. A recent study found that the decrease of miR-363-3p in obese people with metabolic syndrome was associated with the increase of serum cholesterol [13]. Liu et al. found that there were abnormal expressions of five miRNAs in ovarian endometriosis, including miR-363-3p, and their expression in ectopic endometrium group was significantly reduced compared with normal endometrium tissue [14]. Another study showed that the expression of miR-363-3p in plasma exosomes was inconsistent between PCOS patients and healthy people, showing a downregulation trend in the PCOS group [15]. Nevertheless, the current research on the relationship between miR-363-3p and PCOS is still unclear. The purpose of this study was to analyze the expression level of miR-363-3p in PCOS patients, the relationship between miR-363-3p and the patient’s blood biochemical indicators, and the predictive value of miR-363-3p for pregnancy in PCOS patients.

## Methods

### Inclusion of the study population

A total of 126 infertile patients due to PCOS admitted to Huanggang Central Hospital of Yangtze University were selected. The diagnosis of PCOS was in accordance with the 2003 Rotterdam criteria [16]. Inclusion criteria: (1) Rare ovulation and/or anovulation. The standard of rare ovulation is menstrual cycle > 35 days. (2) The clinical manifestations and/or biochemical signs of hyperandrogenism, among which hyperandrogenism is based on the elevated total testosterone level measured in laboratory. The clinical features of hyperandrogenism may be acne and hirsutism (excluding other disorders that can lead to hyperandrogenism, such as congenital adrenal hyperplasia, androgen-secreting tumors, and Cushing’s syndrome). (3) Polycystic ovarian changes: ≥12 follicles with a diameter of 2-9 mm in one or both ovaries, and/or ≥ 10mL in ovarian volume. PCOS can be diagnosed when patients meet any two of the above three criteria. The control group consisted of 110 infertile women caused by male factors, with normal menstruation, no clinical manifestations of androgen excess, normal ovulation and normal morphology of bilateral ovary by ultrasound examination. Exclusion criteria: (1) History of ovarian surgery or radiotherapy and chemotherapy, endometriosis, uterine fibroids, endometrial polyps, uterine malformations, intrauterine adhesions, various genetic diseases, and acute inflammation. (2) No history of hormone medication in the past three months. All subjects were unrelated Han Chinese from the same geographical area. This study was performed in accordance with the principles of the Declaration of Helsinki. The experimental scheme complies with the requirements of the Ethics Committee of Huanggang Central Hospital of Yangtze University. The study is approved by the Ethics Committee of Huanggang Central Hospital of Yangtze University, and the patients and their families signed the informed consent.

During the subject’s natural menstrual cycle or during the period of progesterone-induced withdrawal bleeding in amenorrhea (follicular phase: days 3–5 of menstruation), after a 10-hour overnight fasting period, a 5 mL venous blood sample was drawn the next morning. The serum levels of luteinizing hormone, follicle-stimulating hormone (FSH), prolactin (PRL), estradiol (E2), progesterone (P), total testosterone (TT), fasting blood glucose (FBG) and fasting insulin were measured by chemiluminescence immunoassay. Total cholesterol (TC), triglyceride (TG), high-density lipoprotein cholesterol (HDL-C) and low-density lipoprotein cholesterol (LDL-C) were determined by colorimetry. The homeostasis model of insulin resistance (HOMA-IR) index was used to evaluate the insulin resistance of patients. HOMA-IR = FBG level (mmol/L) ⋅ fasting insulin level (mIU/L) / 22.5.

### Treatment methods and sample collection

All patients received ovulation induction therapy. Clomiphene citrate, 50 mg/d, was taken orally from the third day of menstrual cycle or withdrawal bleeding of progesterone for 5 consecutive days. On the 8th day of menstruation, human menopausal urinary gonadotropin (75 IU/d) was injected intramuscularly, and the duration of administration was determined according to follicular development. When the diameter of the follicle is ≥ 18 mm and the urine LH (+), the intramuscular injection of chorionic gonadotropin is started, and the intercourse was performed during the ovulation. Fasting peripheral venous blood was taken 6–8 days after ovulation, and the supernatant was collected after centrifugation and stored in -80℃ refrigerator for later use.

### Detection of serum miR-363-3p

Reverse transcription quantitative polymerase chain reaction (RT-qPCR) was used to detect the serum level of miR-363-3p. Total RNA in serum was extracted according to the instructions of TRIzol, and the purity of RNA was detected by spectrophotometer. RNA was reverse transcribed into cDNA by reverse transcription kit, and U6 was used as internal reference for amplification. All primers needed were synthesized and provided by GenePharma Company (Nanjing, China). Reaction conditions: 94℃, 3min, 1 cycle; 92℃, 30s, 60℃, 30s, 75℃, 30s, 40 cycles. Primers: miR-363-3p: 5’-CGCAAGAACATCTCCAAG-3’ (forward), 5’-CTCAACTGGTGTCGTGGA-3’ (reverse); U6: 5’-CTCGCTTCGGCAGCACA-3’ (forward), 5’-AACGCTTCACGAATTTGCGT-3’ (reverse). After the reaction, the relative expression of serum miR-363-3p was calculated by 2^−ΔΔCt^ method.

### Pregnancy judgment and follow-up analysis

Serum human chorionic gonadotropin (HCG) was measured on the 14th day after ovulation, and when the HCG level was significantly increased, it was defined as biochemical pregnancy. Clinical pregnancy was confirmed when the presence of a gestational sac was indicated by transvaginal ultrasound on day 30–35 after ovulation. The patients were followed up for one year through the outpatient clinic, and the pregnancy status of the patients was recorded during the follow-up period.

### Statistical analysis

SPSS 20.0 was used for data processing. Counting data are represented by n for Chi-square test; measurement data conforming to normal distribution are represented by (mean ± SD) for *t* test. Pearson test was used for correlation analysis. Logistic regression analysis was used to evaluate the risk factors of pregnancy failure after ovulation induction therapy in PCOS patients.

## Results

### General clinical conditions

The general clinical data and biochemical indicators of the control group and PCOS group are summarized in Table 1. It can be seen from this result that there were no significant differences in age, body mass index (BMI), systolic blood pressure (SDP), diastolic blood pressure (DBP), triglyceride (TG), high-density lipoprotein cholesterol (HDL-C), low-density lipoprotein cholesterol (LDL-C) between control group and PCOS group (*P* > 0.05). Notably, in PCOS group, the levels of fasting blood glucose (FBG), fasting insulin (FINS), homeostasis model of insulin resistance (HOMA-IR), luteinizing hormone, follicle-stimulating hormone (FSH), estradiol (E2), testosterone (T), prolactin (PRL), progesterone (P), anti-mullerian hormone (AMH), and total cholesterol (TC) were significantly higher than those in control group (*P* < 0.05).

**Table 1:** Comparison of general data between PCOS group and control group

Indicators	Controls (n = 110)	PCOS (n = 126)	*P* value
Age (Year)	28.75 ± 2.41	28.64 ± 2.56	0.426
BMI (kg/m^2^)	23.41 ± 2.51	23.96 ± 2.72	0.103
SBP (mm Hg)	118.36 ± 13.14	119.73 ± 12.98	0.108
DBP (mm Hg)	72.48 ± 6.91	74.22 ± 6.82	0.465
FBG (mmol/L)	4.68 ± 0.46	5.01 ± 0.82	0.000
FINS (mIU/L)	7.16 ± 1.54	23.96 ± 4.91	0.000
HOMA-IR	1.57 ± 0.32	2.44 ± 0.95	0.000
LH (IU/L)	5.13 ± 1.75	12.31 ± 5.63	0.000
FSH (IU/L)	6.12 ± 1.81	7.88 ± 1.42	0.000
E_2_ (pmol/L)	173.76 ± 40.51	102.61 ± 45.66	0.000
T (µg/L)	0.43 ± 0.20	0.89 ± 0.23	0.000
PRL (µg/L)	10.57 ± 4.27	13.85 ± 5.24	0.000
P (nmol/L)	0.81 ± 0.41	1.15 ± 0.49	0.000
AMH (ng/mL)	3.42 ± 1.36	9.59 ± 2.11	0.000
TC (mmol/L)	4.60 ± 0.36	4.95 ± 0.33	0.045
TG (mmol/L)	0.95 ± 0.13	0.92 ± 0.21	0.387
HDL-C (mmol/L)	1.60 ± 0.10	1.63 ± 0.08	0.174
LDL-C (mmol/L)	2.56 ± 0.14	2.52 ± 0.18	0.225

Abbreviations: PCOS: polycystic ovary syndrome; BMI: body mass index; SBP: systolic blood pressure; DBP: diastolic blood pressure; FBG: fasting blood glucose; FINS: fasting insulin; HOMA-IR: homeostasis model assessment of insulin resistance; LH: luteinizing hormone; FSH: follicle-stimulating hormone; E_2_: estradiol; T: testosterone; PRL: prolactin; P: progesterone; AMH: anti-mullerian hormone; TC: total cholesterol; TG: triglyceride; HDL-C: high-density lipoprotein cholesterol; LDL-C: low-density lipoprotein cholesterol. Data are presented as mean ± standard deviation (SD).

### Serum mir-363-3p expression level and its correlation with clinical data of PCOS patients

**Fig. 1:**
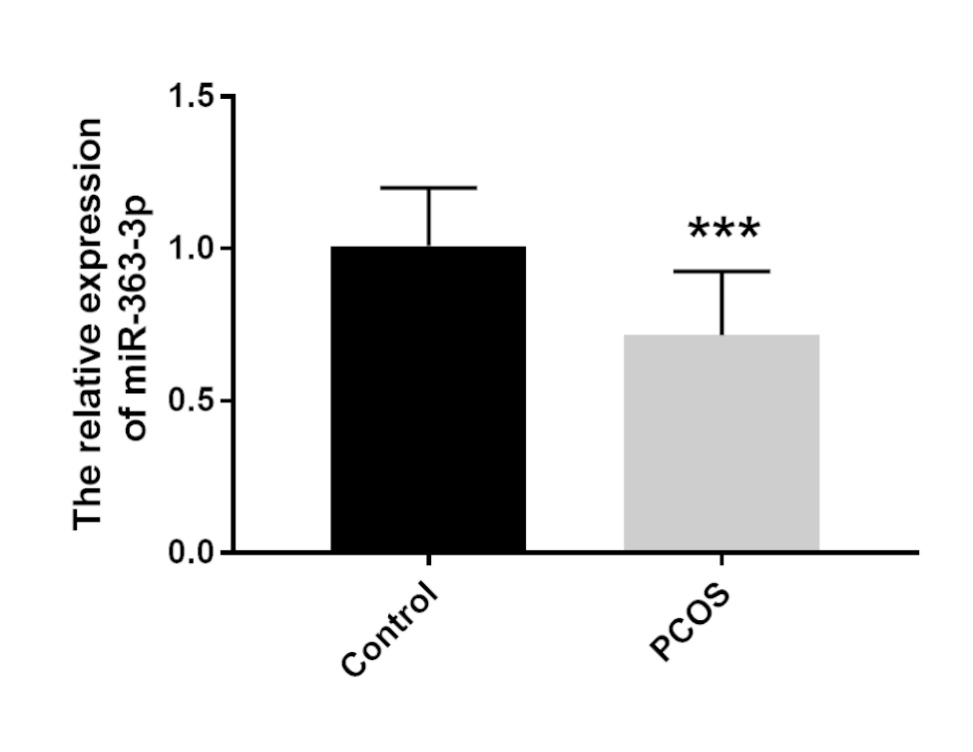
The expression level of serum miR-363-3p in control and PCOS groups. Serum level of miR-363-3p in PCOS group was reduced in comparison to the control group. ^***^P < 0.001

The expression level of serum miR-363-3p in control group and PCOS group was detected by RT-qPCR. Compared with the control group, the relative expression level of miR-363-3p was significantly decreased (Fig. 1, P < 0.001). Additionally, Pearson correlation coefficient revealed that the expression of serum miR-363-3p was negatively correlated with FBG, FINS, HOMA-IR, LH, FSH, E2, T, PRL, P, AMH, TC (Table 2, *P* < 0.05).

**Table 2:** Correlation between serum miR-363-3p level and

Indexes	Correlation with serum miR-363-3p level (r)	*P* value
Age	-0.154	0.515
BMI	-0.311	0.063
SBP	-0.116	0.625
DBP	-0.108	0.713
FBG	-0.604	< 0.001
FINS	-0.593	< 0.001
HOMA-IR	-0.581	< 0.001
LH	-0.635	< 0.001
FSH	-0.670	< 0.001
E_2_	-0.722	< 0.001
T	-0.757	< 0.001
PRL	-0.594	< 0.001
P	-0.732	< 0.001
AMH	-0.559	< 0.001
TC	-0.401	0.031
TG	-0.019	0.862
HDL-C	-0.131	0.606
LDL-C	-0.144	0.533

Abbreviations: PCOS: polycystic ovary syndrome; BMI: body mass index; SBP: systolic blood pressure; DBP: diastolic blood pressure; FBG: fasting blood glucose; FINS: fasting insulin; LH: luteinizing hormone; FSH: follicle-stimulating hormone; E2: estradiol; T: testosterone; PRL: prolactin; P: progesterone; AMH: anti-mullerian hormone; HOMA-IR: homeostasis model assessment of insulin resistance; TC: total cholesterol; TG: triglyceride; HDL-C: high-density lipoprotein cholesterol; LDL-C: low-density lipoprotein cholesterol

### Pregnancy status in PCOS patients after ovulation induction therapy

After systematic ovulation induction treatment, 70 of 126 PCOS patients were successfully conceived, and 56 failed. Patients who successfully conceived were classified as pregnant group, while patients who were not pregnant were classified as non-pregnant group. The clinical information of these two groups is summarized in Table 3. After analysis, it could be seen that the level of BMI, FINS, HOMA-IR, LH, FSH, T, P, and AMH of non-pregnant group is significantly higher than those in pregnant group (*P* < 0.05).

**Table 3:** Comparison of indicators between pregnant group and non-pregnant group in PCOS patients

Indicators	Pregnancy group (n = 70)	Non-pregnancy group (n = 56)	*P* value
Age (Year)	28.39 ± 2.41	28.31 ± 2.49	0.656
BMI (kg/m^2^)	23.51 ± 2.86	23.52 ± 2.56	0.517
SBP (mm Hg)	120.66 ± 11.79	121.64 ± 12.12	0.791
DBP (mm Hg)	73.05 ± 7.08	74.82 ± 6.93	0.153
FBG (mmol/L)	4.99 ± 0.87	5.11 ± 0.81	0.051
FINS (mIU/L)	9.22 ± 1.78	12.96 ± 2.15	0.000
HOMA-IR	2.02 ± 0.21	2.61 ± 0.37	0.000
LH (IU/L)	10.27 ± 2.26	16.02 ± 4.16	0.000
FSH (IU/L)	7.01 ± 2.04	10.22 ± 3.13	0.000
E_2_ (pmol/L)	171.16 ± 106.35	170.81 ± 104.34	0.756
T (µg/L)	0.63 ± 0.15	1.02 ± 0.30	0.000
PRL (µg/L)	13.80 ± 4.75	13.93 ± 5.59	0.891
P (nmol/L)	1.23 ± 0.51	1.06 ± 0.52	0.015
AMH (ng/mL)	6.86 ± 1.59	11.23 ± 2.01	0.000
TC (mmol/L)	4.85 ± 0.34	4.90 ± 0.35	0.261
TG (mmol/L)	0.91 ± 0.20	0.89 ± 0.23	0.279
HDL-C (mmol/L)	1.64 ± 0.11	1.62 ± 0.07	0.356
LDL-C (mmol/L)	2.59 ± 0.21	2.61 ± 0.26	0.217

Abbreviations: PCOS: polycystic ovary syndrome; BMI: body mass index; SBP: systolic blood pressure; DBP: diastolic blood pressure; FBG: fasting blood glucose; FINS: fasting insulin; LH: luteinizing hormone; FSH: follicle-stimulating hormone; E_2_: estradiol; T: testosterone; PRL: prolactin; P: progesterone; AMH: anti-mullerian hormone; HOMA-IR: homeostasis model assessment of insulin resistance; TC: total cholesterol; TG: triglyceride; HDL-C: high-density lipoprotein cholesterol; LDL-C: low-density lipoprotein cholesterol. Data are presented as mean ± standard deviation (SD).

### Expression and diagnostic value of mir-363-3p in pregnancy and non-pregnancy groups

**Fig. 2:**
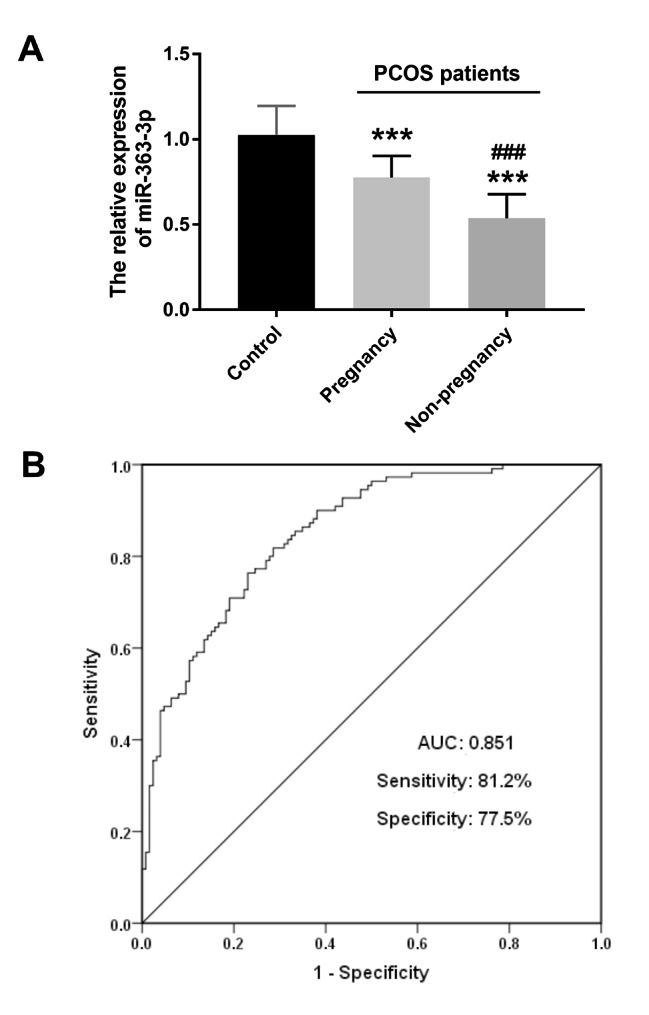
Expression of miR-363-3p in PCOS pregnancy group and non-pregnancy group and its discriminative effect on pregnancy and non-pregnancy. **(A)** Compared with the control group, the miR-363-3p level of both PCOS pregnant group and PCOS non-pregnant group was significantly decreased, while the miR-363-3p level of PCOS non-pregnant group was lower than that of the pregnant group. **(B)** ROC analysis. MiR-363-3p has a good discrimination value between pregnant and non-pregnant people in PCOS patients. ^***^P < 0.001 VS Control group; ^###^*P* < 0.001 VS PCOS pregnant group

As shown in Fig. 2A, compared with the control group, both the pregnant group and the non-pregnant group had significantly lower miR-363-3p levels (*P* < 0.001). In addition, the miR-363-3p levels in the non-pregnancy group was lower than that in the pregnant group (*P* < 0.001). Furthermore, ROC analysis showed that miR-363-3p had a high diagnostic accuracy for distinguishing pregnant women from non-pregnant people in PCOS patients, with the area under the curve (AUC) of 0.851, the sensitivity and specificity of 81.2% and 77.5%, respectively (see Fig. 2B).

### Possible factors influencing pregnancy failure after ovulation induction therapy in PCOS patients

Logistic regression analysis was used to evaluate the influencing factors of pregnancy success in PCOS patients. Table 4 showed that high levels of LH (OR: 2.839, 95% CI: 1.175–6.863, P = 0.020), T (OR: 3.689, 95% CI: 1.872–9.253, P = 0.009), PRL (OR: 2.562, 95% CI: 1.062–6.178, P = 0.036) and low level of miR-363-3p (OR: 0.243, 95% CI: 0.095–0.617, P = 0.003) were independent risk factors for unsuccessful pregnancy in PCOS patients after ovulation induction therapy.

**Table 4:** Factors affecting pregnancy failure in PCOS patients after induction of excretion

Variables	OR	95% CI	*P* value
Age	1.172	0.513–2.677	0.706
BMI	0.821	0.340–1.982	0.660
SBP	2.128	0.855–5.294	0.105
DBP	1.520	0.637–3.629	0.346
FBG	0.708	0.298–1.685	0.435
FINS	1.754	0.738–4.166	0.203
HOMA-IR	0.652	0.269–1.590	0.343
LH	2.839	1.175–6.863	0.020
FSH	2.502	0.974–6.428	0.059
E2	0.946	0.404–2.214	0.899
T	3.689	1.872–9.253	0.009
PRL	2.562	1.062–6.178	0.036
P	1.561	0.684–3.527	0.329
AMH	1.228	0.542–2.782	0.622
TC	0.720	0.296–1.749	0.468
TG	1.345	0.601–3.009	0.490
HDL-C	1.192	0.526–2.705	0.674
LDL-C	1.165	0.494–2.747	0.727
miR-363-3p	0.243	0.095–0.617	0.003

Abbreviations: PCOS: polycystic ovary syndrome; BMI: body mass index; SBP: systolic blood pressure; DBP: diastolic blood pressure; FBG: fasting blood glucose; FINS: fasting insulin; LH: luteinizing hormone; FSH: follicle-stimulating hormone; E_2_: estradiol; T: testosterone; PRL: prolactin; P: progesterone; AMH: anti-mullerian hormone; HOMA-IR: homeostasis model assessment of insulin resistance; TC: total cholesterol; TG: triglyceride; HDL-C: high-density lipoprotein cholesterol; LDL-C: low-density lipoprotein cholesterol

### Comparison of pregnancy outcomes between control and PCOS pregnancy groups

After the follow-up, the pregnancy outcomes and the incidence of related complications were summarized and analyzed. The results suggested that compared with the control group, the incidence of premature delivery, macrosomia and gestational diabetes in the PCOS pregnancy group was significantly enhanced (Table 5, *P* < 0.05).

**Table 5:** Comparison of pregnancy outcomes between control group and pregnancy group

Outcomes	Control group (n = 110)	Pregnancy group (n = 70)
Premature delivery	3 (2.73%)	10 (14.29%) *
Abortion	2 (1.82%)	4 (5.71%)
Gestational diabetes	2 (1.82%)	9 (12.86%) *
Fetal macrosomia	4 (3.64%)	12 (17.14%) *
Gestational hypertension	2 (1.82%)	1 (1.43%)
Low birth weight infant	6 (5.45%)	6 (8.57%)

Note: Data are expressed as n (%). *P < 0.05

## Discussion

PCOS is the most common endocrine disorder syndrome in women of childbearing age, which can not only lead to infertility, but also be associated with insulin resistance, obesity, diabetes and cardiovascular disease [17, 18]. Therefore, the monitoring and evaluating the treatment of PCOS is an important link to reduce the related long-term complications. Despite the long-term and detailed study, it is still difficult to clarify the etiology and pathogenesis of PCOS. At present, the combined effect of genetic and environmental factors is a recognized conclusion of the pathogenesis of PCOS [19]. In recent years, researchers on the etiology of PCOS have begun to pay attention to non-coding RNAs, especially miRNA. MiRNA participates in various biological activities of the body, and its expression is different in pathological conditions such as malignant tumor or endocrine and metabolic diseases. Similarly, miRNAs are involved in the metabolic processes of body substances, including the metabolism and transport of cholesterol and fatty acids, the regulation of pancreas islet function and the metabolism and transport of glucose [20, 21]. The above evidence indicates that miRNAs play an extraordinary role in human physiology and disease.

In this study, the expression level of serum miR-363-3p in PCOS group was significantly lower than that in the control group. The expression levels of serum miR-363-3p in this study were consistent with the results in plasma exosomes of PCOS detected by Jiang et al. [15]. At the same time, the levels of FBG, FINS, HOMA-IR, sex hormones (E2 level was lower than that in the control group) and TC in PCOS group were significantly higher than those in the control group, suggesting that the abnormality of miR-363-3p might be involved in the pathogenesis of PCOS and related to the blood biochemical abnormality of PCOS patients. In 1980, Burghen described the relationship among hyperinsulinemia, insulin resistance (IR) and PCOS for the first time. Statistics show that about 50–70% of PCOS patients would develop into IR [22]. The FBG level of PCOS patients is generally higher than that of the control group. Long-term high-level of FBG can make the blood insulin content increase continuously, thus inducing hyperinsulinemia, further inducing insulin resistance, and ultimately inducing the disorder of glucose metabolism and reproductive hormones [23]. Presently, the correlation between miR-363-3p and insulin resistance has only been verified in animal experiments. Shu et al. found that miR-363-3p expression was significantly reduced in a mouse model of high-fat diet-induced insulin resistance, while resveratrol injection significantly enhanced the expression level of miR-363-3p in the mouse [24]. On this basis, the correlation between miR-363-3p and clinical indicators in PCOS patients was further analyzed, and then it was found that the levels of FBG, FINS, HOMA-IR, sex hormones, AMH and TC were negatively correlated with the level of miR-363-3p, which once again proved the important role of miR-363-3p in PCOS.

Due to rare ovulation, PCOS patients have a low probability of natural pregnancy, so ovulation induction therapy is needed to meet fertility requirements [25]. In this study, after receiving ovulation induction therapy, 70 PCOS patients were successfully pregnant, and 56 others were not pregnant. After further analysis, it was found that compared with the control group, the serum levels of miR-363-3p in pregnant and non-pregnant patients showed a downward trend, while the miR-363-3p levels of non-pregnant group decreased more than that in the pregnant group. Logistic regression analysis suggested that high levels of LH, T, PRL and low level of miR-363-3p in PCOS patients may be independent risk factors for pregnancy failure after ovulation induction therapy. These indicators can predict the pregnancy situation of PCOS patients after ovulation induction therapy to some extent. Furthermore, after a year’s follow-up, it was observed that the proportion of premature delivery, fetal macrosomia, gestational diabetes was significantly higher in PCOS patients than in healthy women. This result may be due to abnormal hormone levels in PCOS patients, which lead to an abnormal uterine environment. Although some PCOS patients can get pregnant, embryo development may also be adversely affected, which requires patients and medical workers to pay more attention and closely monitor the specific conditions of pregnant women and fetuses [26].

There are several limitations in this study that need to be explored. First, studies have found that inflammatory reaction is significantly correlated with the occurrence and development of PCOS [27, 28]. This study did not analyze the inflammatory indicators of the subjects, so the relationship between miR-363-3p and inflammation cannot be verified for the time being. This issue needs to be explored by subsequent experiments. Secondly, this study did not explore the mechanism of miR-363-3p regulating PCOS, for example, the effect of miR-363-3p on ovarian granulosa cells. Thirdly, this study did not analyze the relationship between miR-363-3p and endometrial receptivity. Since endometrial receptivity is related to embryo implantation, this part of the study is indeed important. Finally, in this study, the expression of miR-363-3p in the subjects’ follicular fluid was not collected for analysis, so the relationship between miR-363-3p in peripheral blood and in follicular fluid could not be evaluated at present. In view of the above problems, we believe that in the future scientific research work, we should collect more related literature reports, by referring to the previous research ideas, we can find our own shortcomings and improve the rigor and science of experimental design.

## Conclusions

In this paper, it is considered that the decreased expression of serum miR-363-3p and hormone abnormalities are the characteristics of serological examination in PCOS patients. MiR-363-3p and LH, T, PRL may be the risk factors affecting pregnancy failure in PCOS patients after ovulation induction therapy.

## Data Availability

The datasets used and/or analysed during the current study are available from the corresponding author on reasonable request.
